# C3HeB/FeJ mice with chronic *Mycobacterium avium* complex pulmonary infection exhibit impaired respiratory function but not necrotising granulomatous disease

**DOI:** 10.1186/s44350-025-00004-7

**Published:** 2025-05-15

**Authors:** Timothy David Shaw, Camron M. Pearce, Ha Lam, Ilham M. Alshiraihi, Taru Dutt, Andres Obregon-Henao, Marcella Henao-Tamayo, Mary Jackson, Mercedes Gonzalez-Juarrero

**Affiliations:** 1 Wellcome-Wolfson Institute for Experimental Medicine, School of Medicine, Dentistry and Biomedical Sciences, Queen’s University, Belfast, UK; 2 Mycobacterial Research Laboratories, Department of Clinical Sciences, College of Veterinary Medicine and Biomedical Sciences, Colorado State University, Fort Collins, CO USA; 3 University of Tabuk, Tabuk, Saudi Arabia

**Keywords:** Mycobacterium avium complex, Respiratory infection, Granuloma, Animal model, Plethysmography

## Abstract

**Background:**

*Mycobacterium avium* complex (MAC) is driving a global rise in pulmonary disease (MAC-PD) characterised by chronic infection, granulomatous inflammation and impaired respiratory function. Better animal models are needed to screen candidate therapies targeting bacteria and immune-mediated tissue injury. The C3HeB/FeJ mouse was previously reported to model necrotic granulomatous lung infection in MAC-PD following infection with a low-dose inoculum of the clinical isolate MAC2285R. We investigated whether this model was reproducible with variations in MAC strain and inoculating dose.

**Methods:**

Six-week-old female C3HeB/FeJ mice were infected intratracheally with a clinical isolate of MAC (MAC2285R) or reference strains (MAC104 or MAC101). Mice were culled at 4-weekly intervals post-infection until week 12. Lungs, spleen and liver were harvested for bacterial burden enumeration and histological examination. Whole body plethysmography (WBP) was performed weekly to measure changes in respiratory function (Buxco system).

**Results:**

C3HeB/FeJ mice infected with low dose inoculum of MAC2285R infection exhibited increasing bacterial lung infection for 8 weeks (*p* < 0.05), followed by stable lung burden from weeks 8–12. High dose inoculum resulted in stable lung bacterial burden over 12 weeks. Histological analysis revealed only mild inflammatory changes in both low and high dose inoculum groups at weeks 4, 8 and 12 post-infection, with no evidence of necrotising or non-necrotising granulomatous inflammation. Surrogate measures of respiratory effort (frequency, tidal volume, inspiratory and expiratory flow rates) were increased in mice with high dose inoculum compared to uninfected controls (*p* < 0.001), but not low dose inoculum. Similar findings on lung bacterial burden and histological analysis were found in mice infected with low- and high-dose inoculum of MAC104 and MAC101. MAC104 infection caused greater changes in respiratory function, whereas MAC101 did not significantly affect breathing patterns.

**Conclusion:**

The C3HeB/FeJ mouse is susceptible to chronic MAC infection from intratracheal infection with reference and clinical isolates, but this was not associated with severe granulomatous inflammation as previously reported. A low dose inoculum generated a proliferative lung infection, whereas high dose inoculum resulted in chronic, stable lung bacterial burden. Mice with high-dose inoculum MAC2285R and MAC104 infection also displayed evidence of increased respiratory effort.

**Supplementary Information:**

The online version contains supplementary material available at 10.1186/s44350-025-00004-7.

## Introduction

Mycobacterium avium complex (MAC) is driving a global rise in drug-resistant pulmonary disease (MAC-PD) characterised by chronic infection, granulomatous inflammation and impaired respiratory function [1, 2]. Novel therapeutic strategies are urgently needed, but there is a lack of well-characterised, clinically-relevant models to screen emerging therapies [3].

A spectrum of histopathological findings have been described in surgically-resected lung tissue from patients with MAC-PD, including the appearance of necrotising and non-necrotising granulomatous inflammation [4, 5]. A similar spectrum of disease has been reproduced in immunocompetent mice with chronic MAC-PD using laboratory reference strains and clinical isolates of MAC [6–9]. However, these pathological findings typically appear after > 12 weeks infection and are not sufficiently characterised or standardised for assessing response to host-directed therapies, particularly those that may attenuate necrotising or non-necrotising granuloma formation.

A single report has described rapidly progressive granulomatous MAC-PD with necrosis (within 6 weeks) in the C3HeB/FeJ mouse after infection with the clinical isolate MAC2285R [10]. In this model, C3HeB/FeJ mice were infected by aerosolisation with a low dose inoculum (2500 colony-forming units, CFU) and developed small pulmonary granulomas after 20 days infection that turned necrotic at day 40. This correlated with a rapid proliferation in lung bacterial burden between days 30 and 40. Bacterial burden and pathological changes then appeared to plateau between days 40 and 60, at which point the study ended. It is not known whether these changes are specific to this MAC strain and inoculating dose, nor whether the changes persist beyond 60 days. It is also unknown whether lung pathology in this model correlates with impaired respiratory function.

The aims of our study were to (1) test the replicability of necrotic granulomatous MAC-PD in C3HeB/FeJ mice infected with MAC2285R over a longer (12 week) study, (2) investigate whether these changes were reproducible with different inocula and strains of MAC and (3) use whole-body plethysmography to determine whether C3HeB/FeJ mice with MAC-PD exhibit impaired respiratory function.

## Materials and methods

### Bacteria

The *M. avium* 2285 strain (MAC2285R) with a rough colony morphology and positive for biofilm formation was obtained from a pulmonary MAC patient with a fibrocavitary form of disease (gift from Drs. Stephen Holland and Kenneth Olivier, National Institute of Allergy and Infectious Diseases). *M. avium* subsp. hominissuis strain 101 (MAC101) was isolated in 1983 from human blood and it is also designated as ATCC 700898 [11]. This whole-genome sequenced bacterial strain is the standard strain for MAC susceptibility testing. *M. avium* strain 104 (MAC104) was isolated from an adult AIDS patient in Southern California in 1983 and obtained from ATCC [11]. Stock cultures for each strain were grown at 37 °C in Middlebrook 7H9 liquid medium (HiMedia, M198-500G) supplemented with 10% OADC, 0.5% glycerol, and 0.5% tween 80. Cultures were shaken for 7–14 days to reach exponential growth phase and removed at OD_600_ 0.6–0.8 for experimental use, as previously described [12]. Culture was passed through a 26 ½G needle 15–20 times prior to dilution to minimize clumping.

### Animals

*M. avium* infection was assessed in female C3HeB/FeJ mice. Mice were purchased from the Jackson Laboratories at 6–8 weeks of age. All protocols and use of these animals were approved by the Institutional Animal Care and Use Committee (IACUC) at CSU.

### Infection

Log phase cultures were diluted into sterile 0.9% endotoxin free saline at a concentration of 1 × 10^6^ CFU/mL. Two separate doses of fifty microliters of this bacterial suspension were delivered intratracheally as an intrapulmonary spray instillation to each animal using a high-pressure syringe device (PennCentury), for a targeted dose of 1 × 10^5^ CFU/lung for high dose inoculum or 1 × 10^3^ CFU/lung for low dose inoculum [12]. To confirm the actual bacterial deposition in the lungs, mice (n = 3–4) were sacrificed within 24 h after instillation and their lungs prepared for bacterial quantification.

During infection, the mice were monitored daily for clinical observations (e.g., inactivity, rough fur, hunched posture, increased respiratory rate or effort) and their body weights were taken weekly (see Figure S1 in the online supplement). The remaining groups of mice (*n* = 3–4) were humanely euthanized by CO2 narcosis euthanized at various timepoints and the lungs, spleen, liver were collected from each mouse for further processing and analysis.

### Bacterial burden enumeration

Viable bacteria burden was determined by homogenizing of the lungs, spleen and liver using the Precellys Tissue Homogenizer (Precellys Lysing Kit, 220,325–830). Thereafter, serial fivefold dilutions of each homogenate were plated onto Middlebrook 7H11 agar (Millipore, M0428-500G) containing carbenicillin (Sigma-Aldrich, C1389-1G) and cycloheximide (GoldBio, C-930–10) and subsequently cultured for 14 days at 37ºC until CFU were visible and could be enumerated.

### Histopathology and lesion scoring

The lungs were fixed in 4% paraformaldehyde (PFA) for 48 h then embedded in paraffin for histopathology. Sections from formalin fixed and paraffin embedded (FFPE) tissues were cut at 5 µm, stained with hematoxylin and eosin (H&E) and scanned at 40X magnification using multispectral automated PhenoImager (Akoya Biosciences) for histopathological evaluation. The extent of lung lesion burden was quantified in blinded digital images using an open-source QuPath software for image analysis as described previously [13]. For each tissue section, a region of interest (ROI) was generated at low magnification with a custom tissue detecting algorithm using decision forest training and classification to differentiate tissue versus background based on color and area. Lesions were identified within tissue ROIs at high magnification with an additional custom-made algorithm using decision forest training and classification based on staining intensity, color normalization and deconvolution, area, and morphological features. Percent lesion calculations were integrated into the same algorithm and calculated from tissue area and lesion area as designated by the ROI and lesions detected.

### Whole body plethysmography

Whole Body Plethysmography (WBP) was performed as previously described [14]. WBP measures a range of physiological respiratory parameters in conscious, unrestrained mice [15]. WBP was performed weekly for 12 weeks on both uninfected and infected C3HeB/FeJ mice (*n* = 4). Mice were equilibrated within individual WBP chambers of a Buxco FinePointe Series Whole Body Plethysmograph (Buxco respiratory solutions, Data Sciences International) for 10 min before 10 min of data acquisition. Surrogate measures of respiratory effort and timing were taken, including respiratory frequency (F), tidal volume per breath (TV), maximum inspiratory flow rate (MIFR), maximum expiratory flow rate (MEFR), inspiration time (Ti), end inspiration pause (EIP), expiration time (Te) and end expiration pause (EEP). A 3-point moving average for each parameter was calculated from the mean of the measured point and the preceding two points [14]. Area under the curve (AUC) was calculated for each parameter over the study period for comparative analysis between groups. Data reports were generated in the accompanying FinePointe software and exported to GraphPad Prism v9.5.1 for analysis.

### Statistical analysis

Bacterial burden data were expressed as CFU which were Log10-transformed and analyzed using GraphPad Prism version 9.5.1 (GraphPad software, La Jolla, CA). The statistical analysis was performed using unpaired t-tests (for comparing CFU or WBP data between time points) or ANOVA with Tukey’s multiple comparison test (for comparing AUC analysis on WBP parameters over time).

## Results

### C3HeB/FeJ mice exhibit proliferative infection without granulomatous inflammation in chronic *M. avium* lung disease

**Fig. 1:**
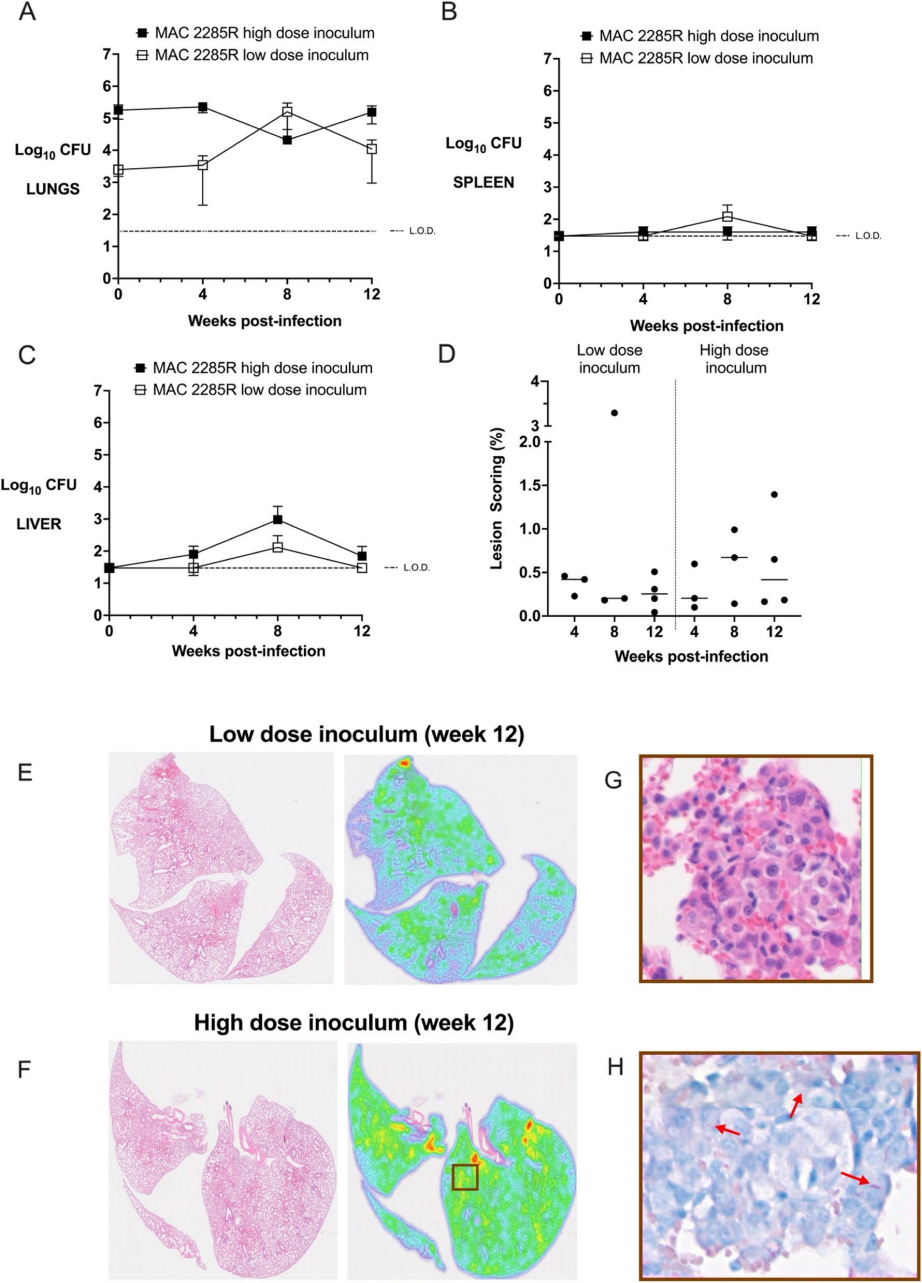
Time course infection of C3HeB/FeJ mice infected with MAC2285R. Lung (**A**), spleen (**B**) and liver (**C**) bacterial burden (y-axis, log_10_ CFU) in C3HeB/FeJ mice infected by intrapulmonary aerosol with high (10^5^ CFU) and low (10^3^ CFU) dose of MAC2285R was measured 4-weekly for 12 weeks. Mice infected with low-dose inoculum exhibited a rise in lung CFU between weeks 4–8 (p < 0.05) with modest extrapulmonary dissemination. Bacterial burden then reduced in lung, spleen and liver between weeks 8–12. Mice infected with high-dose inoculum exhibited largely stable pulmonary bacterial burden over 12 weeks, with liver but not splenic dissemination. Histological lesion scoring of lungs was performed at weeks 4, 8 and 12 (**D**), calculated as the proportion of infected area over the total lung area per animal (n = 3–4 per group). Inflammatory changes were mild, as demonstrated in representative histological heat maps at week 12 post-infection (**E** + **F**). Higher magnification revealed small aggregates of macrophages and epithelioid cells (**G**, **H**&**E** stained) with scattered intracellular bacteria (H, red arrows, Ziehl-Neeson staining). Data is representative of two studies (n = 3–8 per time point) and displayed as mean ± SD (**A**-**C**) or as individual data points with median line (**D**). Differences between time points were analysed by unpaired t-test (**A**-**C**) or by one-way ANOVA with Turkey’s multiple comparison test (D)

C3HeB/FeJ mice infected with low dose inoculum of MAC2285R had stable bacterial burden for the first 4 weeks, followed by a > 1 log-fold rise between weeks 4 and 8 as previously reported (Fig. 1A, *p* < 0.05) [10]. However, lung CFU then fell between weeks 8 and 12, though this did not reach statistical significance (*p* = 0.15). A similar pattern was observed for extrapulmonary dissemination, with detectable splenic and hepatic counts at week 8, but not for week 12 (Fig. 1B + C). By contrast, C3HeB/FeJ mice infected with high dose inoculum of MAC2285R had largely stable bacterial burden between weeks 0 and 12 post-infection (Fig. 1A, *p* = 0.10). Hepatic dissemination occurred earlier (by week 4) and with greater burden, but had started to reduce by week 12 (Fig. 1B). There was no detectable splenic CFUs at any time point (Fig. 1C).

Histopathological analysis of H&E stained lung sections revealed there were mild inflammatory changes, with small foci of aggregated macrophages and epithelioid cells, with scattered intracellular bacilli (Fig. 1D-G). In contrast to previous reports [10], negligible granulomatous inflammation was observable at week 12 post-infection (Fig. 1F + G). Mean lesion score was < 1.5% for both low-dose and high-dose infected mice at all time points, with no significant difference detected between groups (Fig. 1D). Analysis of lung sections from earlier time points (weeks 4 and 8 post-infection) demonstrated similar findings of mild inflammatory injury following low- and high-dose inoculum (Figure S2 in the online supplement). Acid-fast stained bacilli were observed intracellularly and scattered throughout the tissue, occasionally clustered in regions with small aggregates of macrophages and epithelioid cells (Fig. 1H).

Separately, one group of C3HeB/FeJ mice infected with high-dose inoculum of MAC 2285R were observed for 20 weeks (*n* = 3). H&E stained lung sections revealed no evidence of granulomatous inflammation in these mice either (data not shown).

### C3HeB/FeJ mice infected with high-dose inoculum exhibit measurable evidence of increased respiratory effort in chronic *M. avium* lung disease

**Fig. 2:**
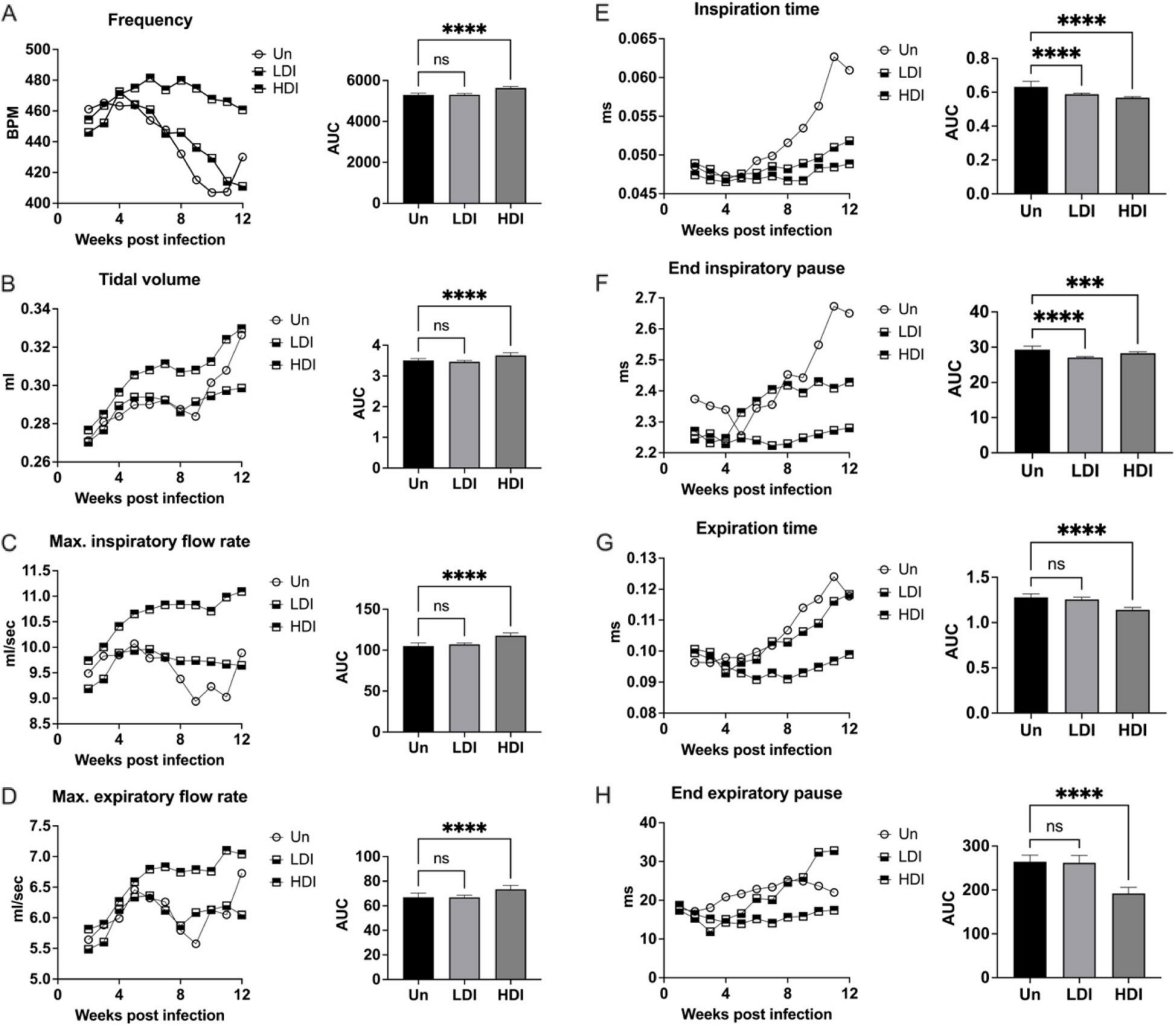
Whole body plethysmography in C3HeB/FeJ mice infected with MAC2285R. Mice infected with high-dose inoculum MAC2285R exhibited evidence of increasing respiratory effort over 12 weeks’, evidence by significantly higher respiratory rate (**A**), tidal volume (**B**), inspiratory flow rate (**C**) and expiratory flow rate (**D**) (*p* < 0.0001 for all). Mice with low dose infection did not exhibit any significant differences in these parameters compared to uninfected mice. MAC infection led to reduced inspiration time (**E**) and end-inspiratory pause (**F**), regardless of infectious dose, but only high dose infection was associated with reduced expiration time (**G**) and end-expiratory pause (**H**) (p < 0.0001). Data points displayed as 3-point moving mean for time-course graphs (with error bars removed for clarity). Data displayed mean + SD for AUC graphs and analysed by one-way ANOVA with Turkey’s multiple comparison test. Data representative of one study with *n* = 4 per group. **** *p* < 0.0001; ns, not significant; Un, uninfected; LDI, low dose inoculum; HDI, high dose inoculum

Whole body plethysmography was performed on C3HeB/FeJ mice infected with low-dose and high-dose inoculum of MAC2285R. Mice with high-dose inoculum exhibited evidence of increasing respiratory effort over 12 weeks compared to uninfected mice, evidenced by significantly higher respiratory rate, tidal volume, maximum inspiratory flow rate and maximum expiratory flow rate (Fig. 2A-D, *p* < 0.001 for all). In contrast, mice with low dose infection did not exhibit any significant differences in these parameters. MAC2285R infection also led to significantly reduced inspiration time (Fig. 2E) and end-inspiratory pause (Fig. 2F), regardless of infectious dose (*p* < 0.001 for both groups), but only high dose infection was additionally associated with reduced expiration time (Fig. 2G) and end-expiratory pause (Fig. 2H) (*p* < 0.001). These changes suggest that the persistent infection caused by high dose inoculum of MAC2285 over 12 weeks correlates with increased respiratory frequency and effort.

### C3HeB/FeJ mice infected with other MAC strains exhibit variations in bacterial control and respiratory function, but no progressive granulomatous disease

We had found that high dose inoculum of MAC2285R caused stable pulmonary infection with impaired respiratory function, whereas low dose inoculum of MAC2285R generated initial proliferative infection followed by plateau, which did not appear to affect respiratory function. In contrast with previous reports [10], MAC2285R did not generate granulomatous lung inflammation. We then proceeded to investigate whether these effects were specific to MAC strain and inocula by repeating the study with two other virulent MAC strains: MAC104 and MAC101. Both strains were isolated from blood of patients with disseminated disease and have become standard strains for MAC susceptibility testing and laboratory disease modelling.

**Fig. 3:**
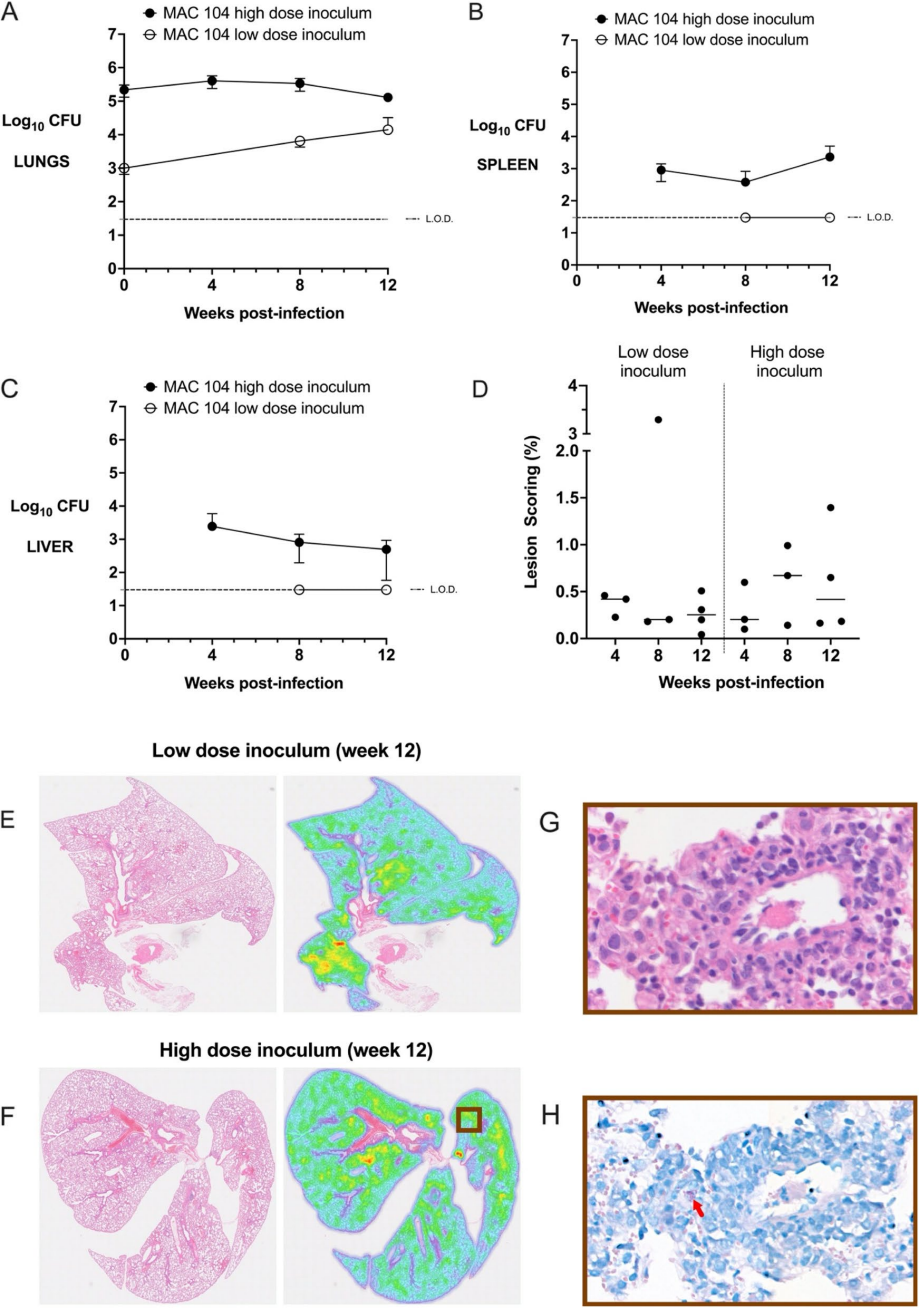
Time course infection of C3HeB/FeJ mice infected with MAC104. Lung (**A**), spleen (**B**) and liver (**C**) bacterial burden (y-axis, log_10_ CFU) in C3HeB/FeJ mice infected by intrapulmonary aerosol with high (10^5^ CFU) and low (10^3^ CFU) dose of MAC101 was measured 4-weekly for 12 weeks (with the exception of week 4 in the low-dose inoculum group for which there were insufficient mice for analysis). Low-dose inoculum led to a proliferative lung infection over 8 weeks (*p* < 0.01), followed by plateau. There was no detectable splenic or hepatic dissemination. High dose inoculum generated a stable pulmonary infection and extrapulmonary dissemination for the first 8 weeks, with a modest reduction in lung bacterial burden between weeks 8–12 (*p* = 0.05). Histological lesion scoring of lungs was performed at weeks 4, 8 and 12 (**D**), calculated as the proportion of infected area over the total lung area per animal (*n* = 3–4 per group). Inflammatory changes were mild, as demonstrated in representative histological heat maps at week 12 post-infection (**E** +** F**). Higher magnification revealed small aggregates of macrophages and epithelioid cells (**G**, **H**&**E** staining) with occasional clusters of intracellular bacteria (H, red arrow, Ziehl-Neeson staining). Data is representative of two studies (*n* = 3–8 per time point) and displayed as mean ± SD (A-C) or as individual data points with median line (**D**). Differences between time points were analysed by unpaired t-test (**A**-**C**) or by one-way ANOVA with Turkey’s multiple comparison test (**D**)

C3HeB/FeJ mice infected with low dose inoculum of MAC104 had proliferative lung infection (approximately 1 log increase over first 8 weeks, *p* < 0.01, Fig. 3A) that stabilised between weeks 8 and 12 (*p* = 0.94). However there was no extrapulmonary dissemination observed at any time point (Fig. 3B + C). High dose inoculum resulted in stable lung bacterial burden for the first 8 weeks (*p* = 0.40), with evidence of reducing burden by week 12 (*p* = 0.05). These mice had detectable splenic and hepatic CFUs from week 4 post-infection which were stable at week 12 (*p* = 0.97). Similarly to mice infected with MAC2285R, histological analysis showed only mild inflammatory changes in mice with low dose or high dose MAC104 inoculum at week 12 (Fig. 3D-G), as well as earlier time points (Figure S3 in the online supplement). Mean lesion score was < 1.0% for low-dose and < 0.5% for high-dose infected mice at all time points, with no significant difference detected between groups. Intracellular bacilli were observed scattered throughout the lung tissue in small clusters (Fig. 3H).

**Fig. 4:**
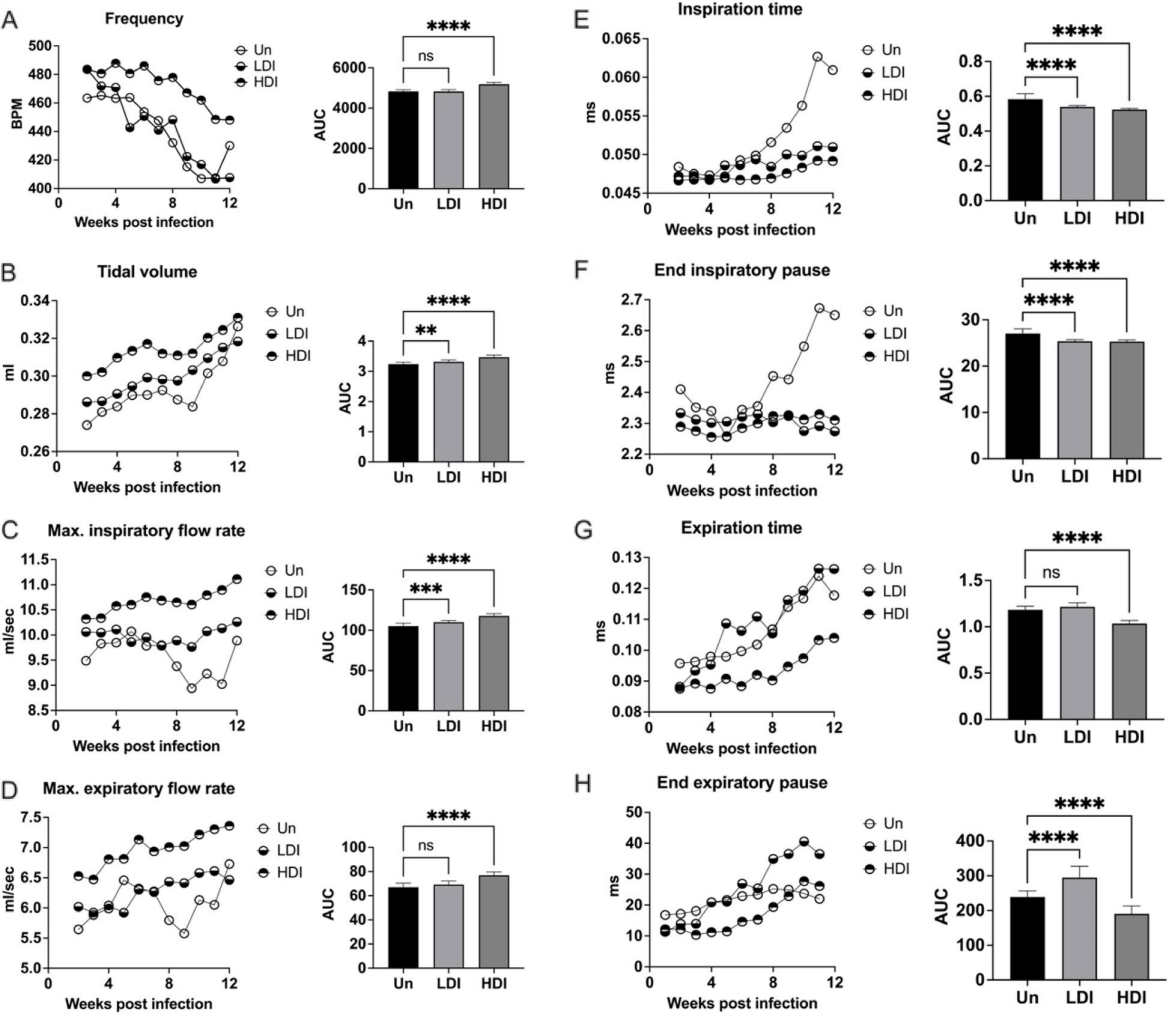
Whole body plethysmography in C3HeB/FeJ mice infected with MAC104. Mice infected with high-dose inoculum MAC104 exhibited evidence of increasing respiratory effort over 12 weeks’, evidence by significantly higher respiratory rate (**A**), tidal volume (**B**), inspiratory flow rate (**C**) and expiratory flow rate (**D**) (*p* < 0.0001). These mice also had significantly reduced inspiration time (**E**), end-inspiratory pause (**F**), expiration time (**G**) and end-expiratory pause (**H**) (*p* < 0.0001). Mice with low dose infection had similar respiratory response, but less pronounced. Data points displayed as 3-point moving mean for time-course graphs (with error bars removed for clarity). Data displayed mean + SD for AUC graphs and analysed by one-way ANOVA with Turkey’s multiple comparison test. Data representative of one study with *n* = 4 per group. ** *p* < 0.01; *** *p* < 0.001; **** *p* < 0.0001; ns, not significant; Un, uninfected; LDI, low dose inoculum; HDI, high dose inoculum

Whole body plethysmography showed mice infected with high dose inoculum MAC104 exhibited evidence of increasing respiratory effort over 12 weeks compared to uninfected controls, with significantly higher respiratory rate, tidal volume, inspiratory flow rate and expiratory flow rate (Fig. 4) (all *p* < 0.001). These mice also had significantly reduced inspiration time, end-inspiratory pause, expiration time and end-expiratory pause (all *p* < 0.001). Mice with low dose MAC104 infection had a similar respiratory response, but less pronounced.

**Fig. 5:**
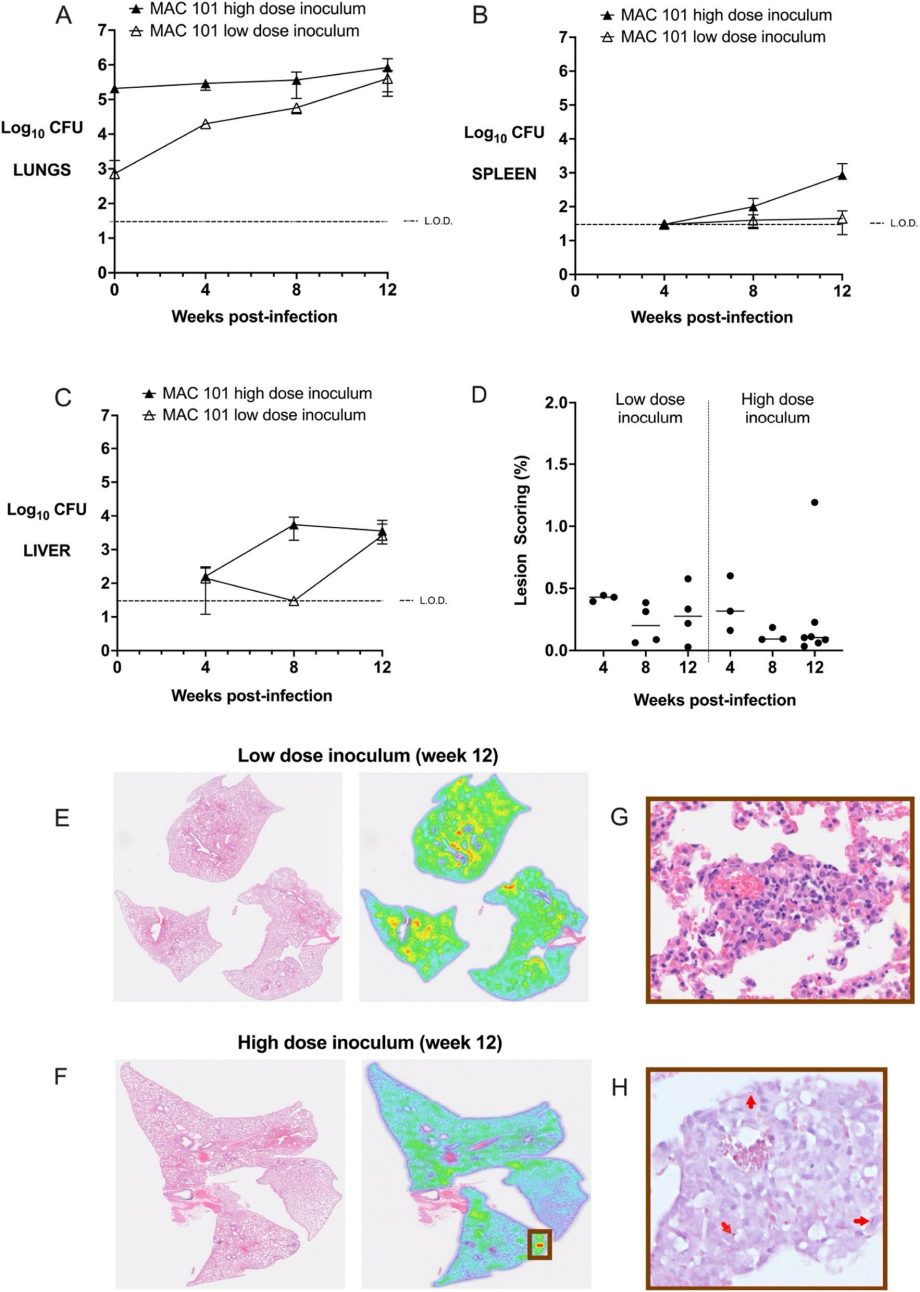
Time course infection of C3HeB/FeJ mice infected with MAC101. Lung (**A**), spleen (**B**) and liver (**C**) bacterial burden (y-axis, log_10_ CFU) in C3HeB/FeJ mice infected by intrapulmonary aerosol with high (10^5^ CFU) and low (10^3^ CFU) dose of MAC101 was measured 4-weekly for 12 weeks. Histological lesion scoring of lungs was performed at weeks 4, 8 and 12 (**D**), calculated as the proportion of infected area over the total lung area per animal (*n* = 3–4 per group). Inflammatory changes were mild, as demonstrated in representative histological heat maps at week 12 post-infection (**E** + **F**). Higher magnification revealed small aggregates of macrophages and epithelioid cells (**G**, **H**&**E** staining) with scattered intracellular bacteria (H, red arrows, Ziehl-Neeson staining). Data is representative of two studies (*n* = 3–8 per time point) and displayed as mean ± SD (**A**-**C**) or as individual data points with median line (**D**). Differences between time points were analysed by unpaired t-test (**A**-**C**) or by one-way ANOVA with Turkey’s multiple comparison test (**D**)

In C3HeB/FeJ mice infected with low dose inoculum of MAC101, a rapidly proliferative lung infection was observed over 12 weeks (approximately 2 log-fold increase, *p* < 0.001, Fig. 5A) with eventual hepatic but not splenic dissemination (Fig. 5B + C). High dose inoculum resulted in a slowly proliferative lung bacterial infection over 12 weeks (*p* < 0.05), with both hepatic and splenic dissemination from week 8 (Fig. 5A-C). As with other MAC strains, histological analysis showed mild inflammation only in mice with low or high dose inoculum at any time point (Fig. 5D-G). Mean lesion score was < 0.5% for both low-dose and high-dose infected mice at all time points, with no significant difference detected between groups, and again the intracellular bacilli was observed scattered throughout the lung tissue (Fig. 5H).

**Fig. 6:**
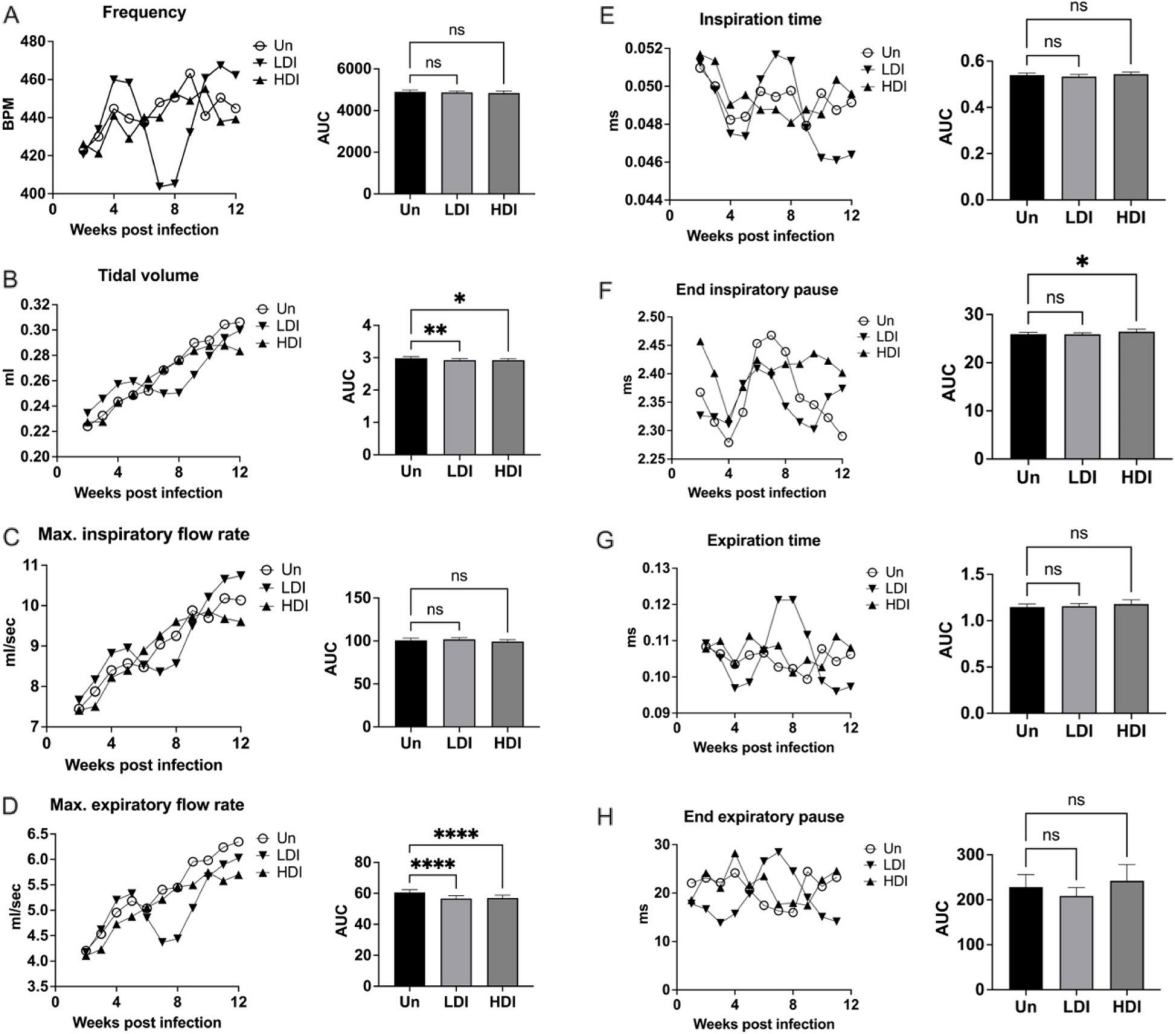
Whole body plethysmography in C3HeB/FeJ mice infected with MAC101. Mice infected with high-dose inoculum MAC101 did not exhibit evidence of increased respiratory effort compared to uninfected mice, as measured by respiratory rate (**A**), tidal volume (**B**), inspiratory flow rate (**C**) or expiratory flow rate (**D**). Neither was there significant difference in inspiration time (**E**), end-inspiratory pause (**F**), expiration time (**G**) or end-expiratory pause (**H**). In contrast, mice with low dose infection exhibited a period of reduced respiratory frequency, tidal volume and expiratory flow rates between weeks 5–10 post-infection (*p* < 0.05), which then recovered to levels comparable with uninfected controls by week 12. Data points displayed as 3-point moving mean for time-course graphs (with error bars removed for clarity). Data displayed mean + SD for AUC graphs and analysed by one-way ANOVA with Turkey’s multiple comparison test. Data representative of one study with *n* = 4 per group. * *p* < 0.05; ** *p* < 0.01; **** *p* < 0.0001; ns, not significant; Un, uninfected; LDI, low dose inoculum; HDI, high dose inoculum

In contrast to MAC2285R and MAC104, whole body plethysmography showed mice infected with high dose inoculum MAC101 exhibited no significant difference in respiratory function compared to uninfected controls over 12 weeks (Fig. 6). In contrast, mice with low dose MAC101 infection had depressed respiratory function (reduced frequency, tidal volume and expiratory flow rates) between weeks 5–10 post-infection (*p* < 0.05). However, these parameters then recovered to levels comparable with uninfected controls by week 12.

## Discussion

Surgical resection of MAC-infected lung tissue from human patients has revealed a heterogenous spectrum of histopathological features. In one case series, consistent findings included destruction of bronchial cartilage and smooth muscle layers, characterised by extensive submucosal infiltration of macrophages, lymphocyte aggregates and epithelioid cells [4]. These infiltrations transformed into granulomas causing ulceration of the bronchial mucosal (necrotic in some cases) and narrowing of the bronchial lumen. Comparative immunohistopathological staining between necrotic and non-necrotic granuloma revealed an association between presence of an outer layer of myofibroblasts and caseating necrosis [16]. A larger study of 356 cases revealed granulomatous disease in the airways and/or parenchyma of resected lung tissue in 42.3% of MAC cases, of which around ¾ also exhibited necrotising granulomas [5]. Therefore, developing reproducible murine models of MAC-PD that capture the spectra of human-like necrotising and non-necrotising granulomatous inflammation is important for the translation of novel antimicrobial and host-directed therapies.

The previous report of pulmonary MAC infection in the C3HeB/FeJ mouse [10] was notable for its rapid development of necrotic granulomatous lung disease, observed around 6 weeks after aerogenic infection with the clinical isolate MAC 2285R. By contrast, it typically requires 16 weeks infection before necrotising granulomatous disease is observed in other immunocompetent mouse models of MAC-PD [17–19]. To date, only one further study has reported on C3HeB/HeJ mice with MAC-PD, generated through aerosol delivery of 10^8.5^ CFU of MAC101 [20]. From weeks 4–8 post-infection, mice received the prodrug SPR720 (a novel aminobenzimidazole) or antimicrobial control (clarithromycin ± ethambutol and rifabutin) via daily oral gavage. MAC 101 lung burden increased modestly over the 8 week study in untreated mice, but fell by > 2 log-fold reductions in all groups treated with maximum doses of therapy. However, the report did not contain any descriptions of histological examination in treated or untreated mice. Therefore, our study is the second to describe histopathological findings in the C3HeB/FeJ mouse with MAC-PD, and the first to perform comparative studies with three MAC strains at both low- and high-dose inoculum.

In contrast to the previous report [10], we did not observe necrotising or non-necrotising granulomatous inflammation in C3HeB/FeJ mice infected with MAC 2285R at 4, 8, 12 or 20 weeks post-infection. The inclusion of three strains of MAC given at two different inocula was a strength of this longitudinal study. However, a corresponding limitation was that numbers of animals at each timepoint was small (3–4 per group), increasing the risk of false negative findings, especially in histopathological analysis. Beyond this, the reasons for the discrepancy between our study and the previous report are unclear: although our methodologies were similar, it is possible some minor variations influenced the outcome. For instance, we infected via intratracheal route, rather than using an aerosoliation chamber and used a low-dose inoculum of approximately 1250 CFU, rather than 2500 CFU. There may also have been methodological differences in bacterial culture, such as concentration of Tween 80 detergent, which affected mycobacterial virulence. However, we have found these three MAC isolates generate severe pulmonary inflammation in balb/c mice under the same culture conditions (unpublished data). Another possible explanation, which we offer cognisant of the limitations of our own findings, is that the previous study may have been influenced by sampling or reporting bias, as it did not specify the number of mouse lungs included in the histological analysis.

After demonstrating the C3HeB/FeJ mouse did not produce necrotic pulmonary granulomas during MAC 2285R infection, we repeated the study using two bacterial reference strains: MAC101 and MAC104. We choose these isolates as they are pathogenic against humans and mice, and are widely available as bacterial reference strains [11]. MAC101 and MAC104 reportedly behave similarly in vivo, generating an infection characterised by stable bacterial burden and granuloma formation in chronically infected C57BL/6 mice [11]. Interestingly, we noted some important inter-strain differences during their infection in the C3HeB/FeJ mouse: only MAC 104 infection with high-dose inoculum resulted in increased respiratory effort, even though MAC 101 lung burden increased more over the 12 week study. It would be useful to see whether these inter-strain differences occur in a different mouse strain, particularly those that are susceptible to granuloma formation.

The C3HeB/FeJ mouse is commonly used in preclinical studies of pulmonary tuberculosis, in which its sst1 allele confers susceptibility to progressive, necrotic granulomatous disease. Taken together, our data suggests the sst1 allele does not confer the same susceptibility to necrotising granuloma formation in MAC pulmonary infection. These findings are consistent with another report, in which the immunocompetent B6.Sst1S mouse (C56BL/6 background mice with the sst1 allele, as well as variant alleles at Scl11a1 and MHC class II) exposed to aerogenic MAC101 exhibited severe histological lung injury after 12 weeks post-exposure, but did not develop necrotic granulomas [8].

Indeed, the development of necrotic granulomas in immunocompetent mice appears to be MAC- and mouse-strain specific, and dependent the chronicity of infection. Caseating granulomas with central necrosis after 16 weeks infection via inhalation with the virulent MAC25291 has been reported in C57BL/6 [17–19]. However, other MAC strains appear to generate non-necrotic granulomatous lung inflammation in C57BL/6 mice, even after 20 weeks infection, as reported for MAC101 [8, 21], MAC15769 [21] and MAC2447 [22]. Balb/c mice are an alternative immunocompetent mouse used in preclinical testing of novel MAC therapies, but also exhibit a spectrum of responses to reference and clinical MAC isolates. Pulmonary infection with some clinical isolates generate only mild granulomatous disease which resolves within 16 weeks along with bacterial clearance [23], whereas others exhibit mature granulomas by weeks 8–16 post-infection [24–26]. Even the MAC25291 strain, which produces necrotic granulomatous lung disease in C57BL/6 mice, appears to generate only non-necrotising granulomatous disease in Balb/c mice after 20 weeks infection [27]. The characterisation of bacterial burden and pathological changes from a variety of MAC and mouse strain combinations would help standardise models for testing therapies and represent a major contribution to the field.

Plethysmography represents an under-utilised preclinical methodology for assessing response to disease and therapy in chronic infection. This is the first report to describe WBP findings in mice with MAC-PD and offers new insight into MAC-specific changes in respiratory function. We previously described WBP findings in pulmonary *M. abscessus* infection, comparing changes in the βENaC transgenic mice with those of C57BL/6 mice as their wild-type background control strain [14]. We found *M. abscessus*-infected C57BL/6 mice had similar respiratory frequency but reduced tidal volume and increased airway resistance compared to uninfected controls, which persisted for 60 days despite complete spontaneous lung bacterial clearance by day 30 post-infection. In contrast, βENaC mice exhibited increased respiratory frequency and tidal volume for the first 15 days post-infection, followed by a dramatic fall in both parameters between days 15 and 60. These changes correlated with extensive pulmonary inflammatory injury, suggesting an initial phase characterised by physiological response to infection, followed by decompensating respiratory function in advanced disease. In our MAC-PD study, the WBP findings of increased respiratory frequency, tidal volume and expiratory flow rate (particularly with high-dose inoculum) were generally consistent with physiological response to a chronic lung infection, but without extensive histopathological injury that would lead to decompensating respiratory function. These changes appeared to be gradual and progressive, in contrast to the sudden deterioration seen in *M. abscessus* infection. An extended study would help to determine whether chronic MAC-PD would eventually lead to sudden or gradual decompensation of respiratory function. In addition, our findings indicate that measures of respiratory effort can be increased in chronic infection, even without significant tissue pathology. We propose these physiological changes may be driven by the mild pulmonary infiltrates of inflammatory cells seen on histology, bringing a rise in local and systemic pro-inflammatory cytokines. These may promote cognitive, neural and/or airway sensitivity resulting in increased respiratory effort. Future studies should explore this with measures of cytokines and soluble inflammatory mediators in the lung and serum. We suggest plethysmography could be incorporated into preclinical models of MAC-PD and other chronic lung infections, to characterise longitudinal changes in respiratory function changes and responses to therapeutic interventions.

## Conclusion

Our data suggests that intrapulmonary infection with high-dose inoculum with MAC generated a stable, chronic infection in the C3HeB/FeJ mouse, with evidence of increased respiratory effort. Low-dose inoculum generated a proliferative lung infection, though with less pronounced changes in respiratory function. However, we found MAC-PD in the C3HeB/FeJ mouse generated, at most, only modest lung inflammation across three different strains of MAC at low- and high-dose inoculum. We propose the C3HeB/FeJ mouse may not consistently recapitulate necrotising granulomatous inflammation, limiting its utility in modelling severe forms of MAC-PD.

## Supplementary Information

Supplementary Material 1: Figure S1. Weight change in mice with pulmonary MAC infection. Figure S2. Histological lung changes in C3HeB/FeJ mice with MAC2285R lung infection. Figure S3. Histological lung changes in C3HeB/FeJ mice with MAC104 lung infection. Figure S4. Histological lung changes in C3HeB/FeJ mice with MAC101 lung infection.

## Data Availability

The datasets used and/or analysed during the current study are available from the corresponding author on reasonable request.

## Abbreviations

AUC: Area under the curve
CFU: Colony-forming unit
EIP: End inspiration pause
EEP: End expiration pause
F: Respiratory frequency
FFPE: Formalin fixed and paraffin embedded
H&E: Hematoxylin and eosin
IACUC: Institutional Animal Care and Use Committee
PFA: Paraformaldehyde
MAC: *Mycobacterium avium* Complex
MAC-PD: MAC pulmonary disease
MIFR: Maximum inspiratory flow rate
MEFR: Maximum expiratory flow rate
ROI: Region of interest
Te: Expiration time
Ti: Inspiration time
TV: Tidal volume per breath
WBP: Whole body plethysmography
