# Supplementary material for: C3HeB/FeJ mice with chronic Mycobacterium avium complex pulmonary infection exhibit impaired respiratory function but not necrotising granulomatous disease
Source: Mycobacteria. 2025 May 15;1(1):4. doi: 10.1186/s44350-025-00004-7 (PMC12081485; doi:10.1186/s44350-025-00004-7)
Supplement: Supplementary file 1 — Supplementary Material 1: Figure S1. Weight change in mice with pulmonary MAC infection. Figure S2. Histological lung changes in C3HeB/FeJ mice with MAC2285R lung infection. Figure S3. Histological lung changes in C3HeB/FeJ mice with MAC104 lung infection. Figure S4. Histological lung changes in C3HeB/FeJ mice with MAC101 lung infection. [file 44350_2025_4_MOESM1_ESM.pdf]

Online Supplement

C3HeB/FeJ mice with chronic *Mycobacterium avium* complex pulmonary infection exhibit impaired respiratory function but not necrotising granulomatous disease

Timothy David Shaw[1,2], Camron M Pearce[2], Ha Lam[2], Ilham M Alshiraihi[2,3], Taru Dutt[2], Andres Obregon-Henao[2], Marcella Henao-Tamayo[2], Mary Jackson[2], Mercedes Gonzalez-Juarrero[2]

[1] Wellcome-Wolfson Institute for Experimental Medicine, School of Medicine, Dentistry and Biomedical Sciences, Queen's University Belfast, UK

[2] Mycobacterial Research Laboratories, Dept. of Clinical Sciences, College of Veterinary Medicine and Biomedical Sciences, Colorado State University, Fort Collins, Colorado, USA

[3] University of Tabuk, Tabuk, Saudi Arabia

A

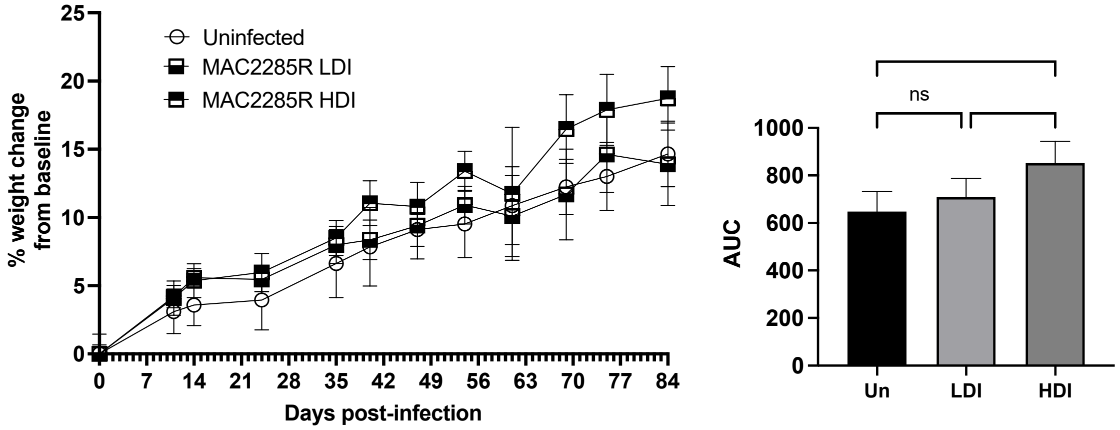
Uninfected
MAC2285R LDI
MAC2285R HDI
% weight change from baseline
Days post-infection
0
7
14
21
28
35
42
49
56
63
70
77
84
25
20
15
10
5
0
ns
AUC
1000
800
600
400
200
0
Un
LDI
HDI

B

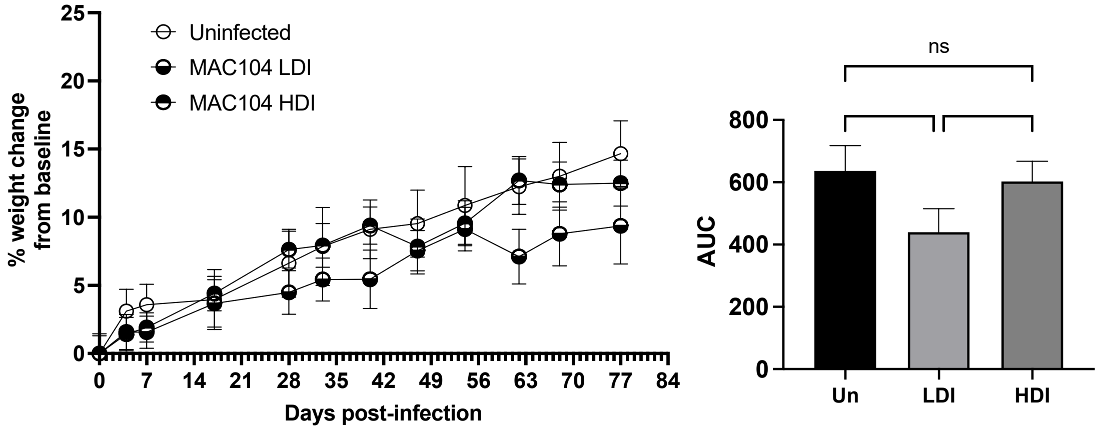
Uninfected
MAC104 LDI
MAC104 HDI
% weight change from baseline
Days post-infection
0
7
14
21
28
35
42
49
56
63
70
77
84
25
20
15
10
5
0
ns
AUC
800
600
400
200
0
Un
LDI
HDI

C

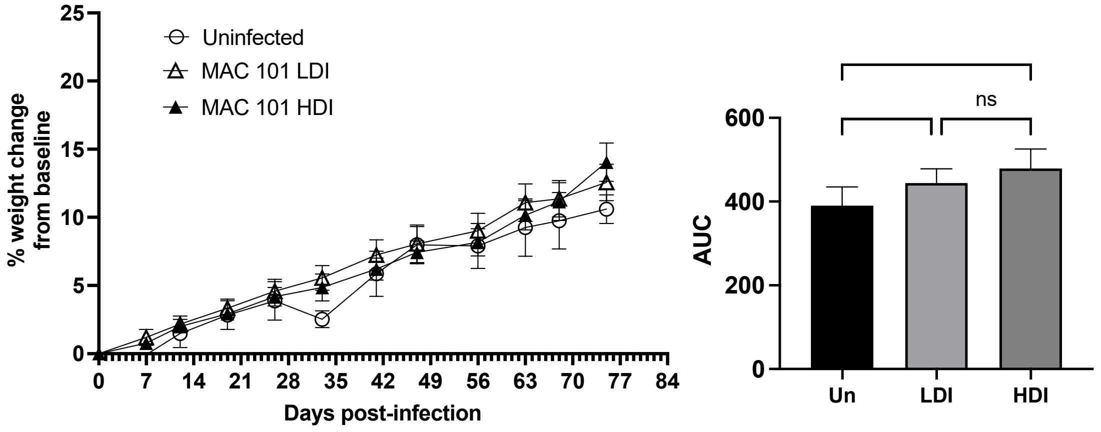
Uninfected
MAC 101 LDI
MAC 101 HDI
% weight change from baseline
Days post-infection
0
7
14
21
28
35
42
49
56
63
70
77
84
25
20
15
10
5
0
ns
AUC
600
400
200
0
Un
LDI
HDI

Figure S1: weight change in mice with pulmonary MAC infection

Over 12 weeks, mice with MAC2285R pulmonary infection had similar or increased rates of weight gain compared to uninfected controls (A). In contrast, mice infected with MAC104 gained weight, but at a reduced rate compared to uninfected controls ($p<0.001$ for both) (B). MAC101 infection was associated with modestly increased weight gain compared to uninfected controls (C). Data displayed mean + SD for time course and AUC graphs and analysed by one-way ANOVA with Turkey's multiple comparison test. Data representative of one study with n≧4 per group. . ** $p<0.01$; *** $p<0.001$; **** $p<0.0001$; ns, not significant; Un, uninfected; LDI, low dose inoculum; HDI, high dose inoculum.

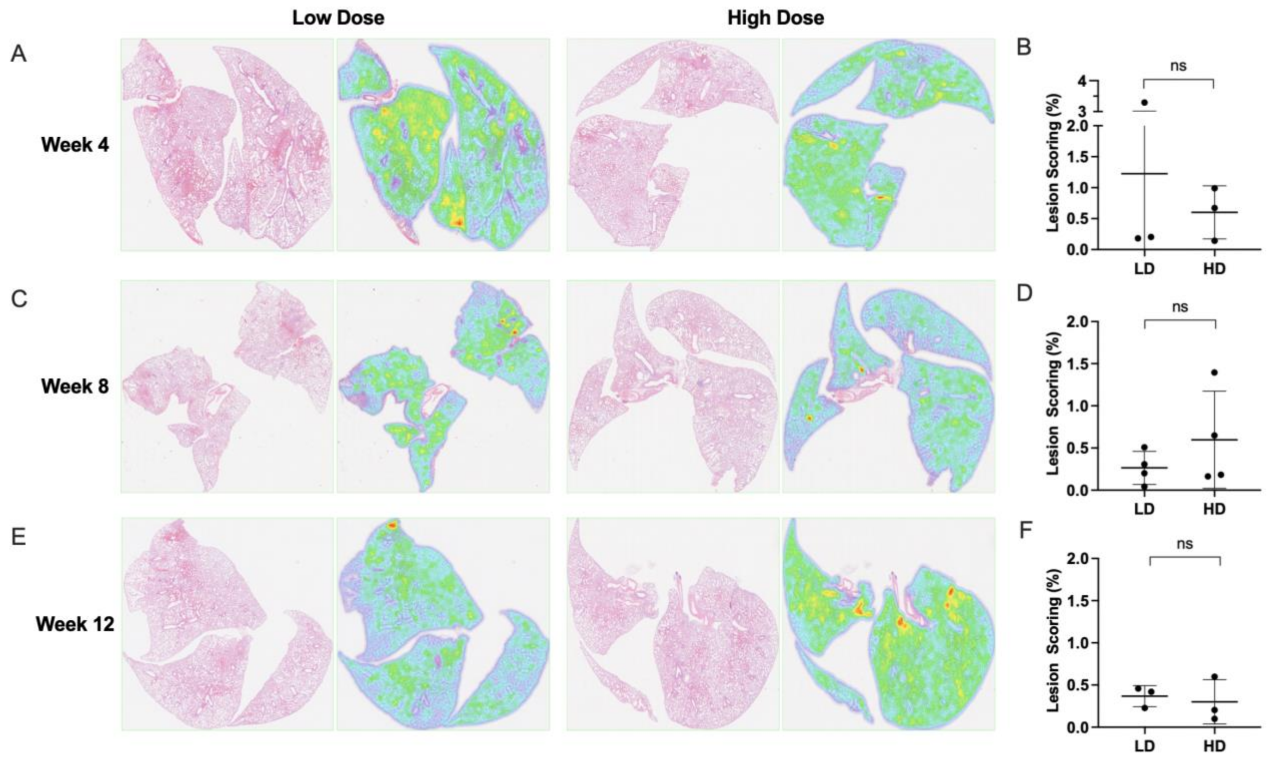

Figure S2: Histological lung changes in C3HeB/FeJ mice with MAC2285R lung infection

Histological heat maps and lesion scoring of lungs at weeks 4 (A+B), weeks 8 (C+D) and weeks 12 (E+F) post-infection with low dose (LD) or high dose (HD) inoculum. Lesion scores were calculated as the proportion of infected area over the total lung area per animal (n=3-4 per group). Data displayed as mean + SD for each time point and analysed by unpaired test. Data representative of two studies with n=3-4 per time point. LD, low dose inoculum; HD, high dose inoculum; ns, not significant.

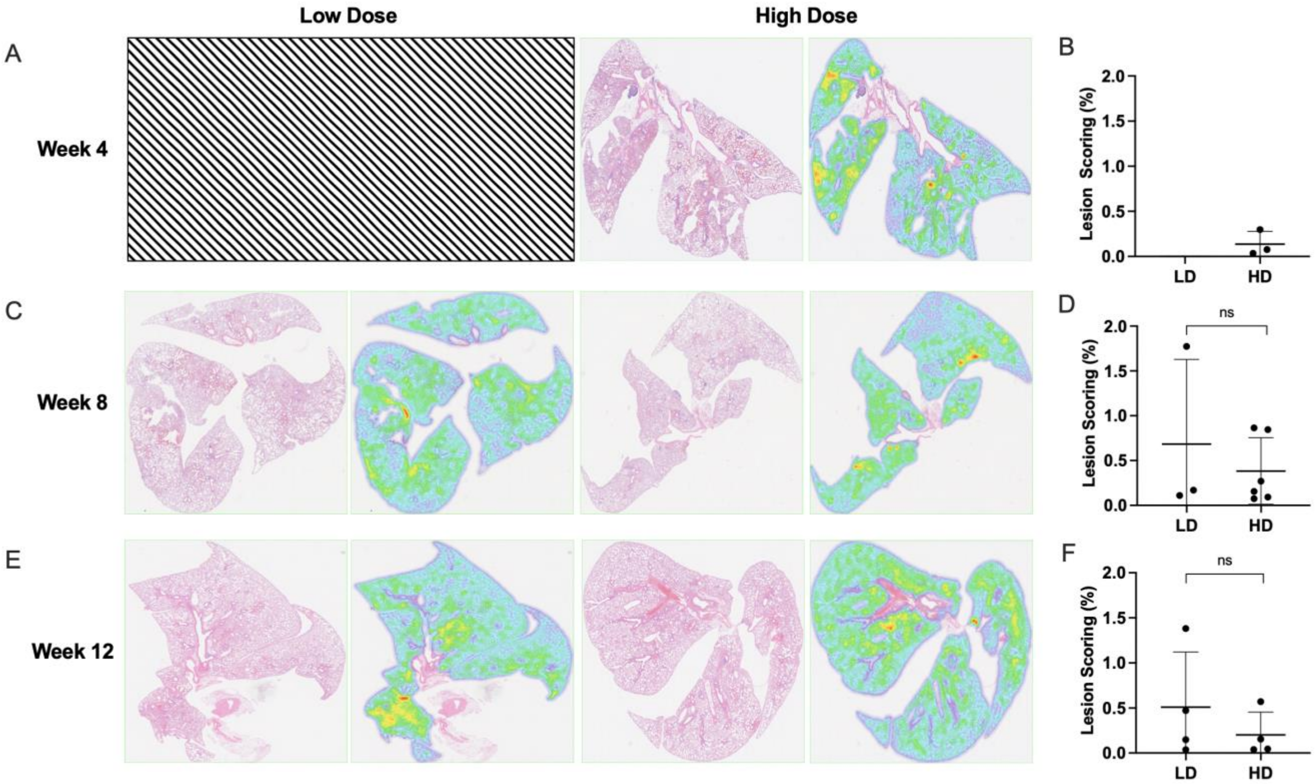

Figure S3: Histological lung changes in C3HeB/FeJ mice with MAC104 lung infection

Histological heat maps and lesion scoring of lungs at weeks 4 (A+B), weeks 8 (C+D) and weeks 12 (E+F) post-infection with low dose (LD) or high dose (HD) inoculum. Data is missing in the low-dose inoculum group for week 4 as there were insufficient mice for analysis. Lesion scores were calculated as the proportion of infected area over the total lung area per animal (n=3-6 per group). Data displayed as mean + SD for each time point and analysed by unpaired test. Data representative of two studies with n=3-6 per time point. LD, low dose inoculum; HD, high dose inoculum; ns, not significant.

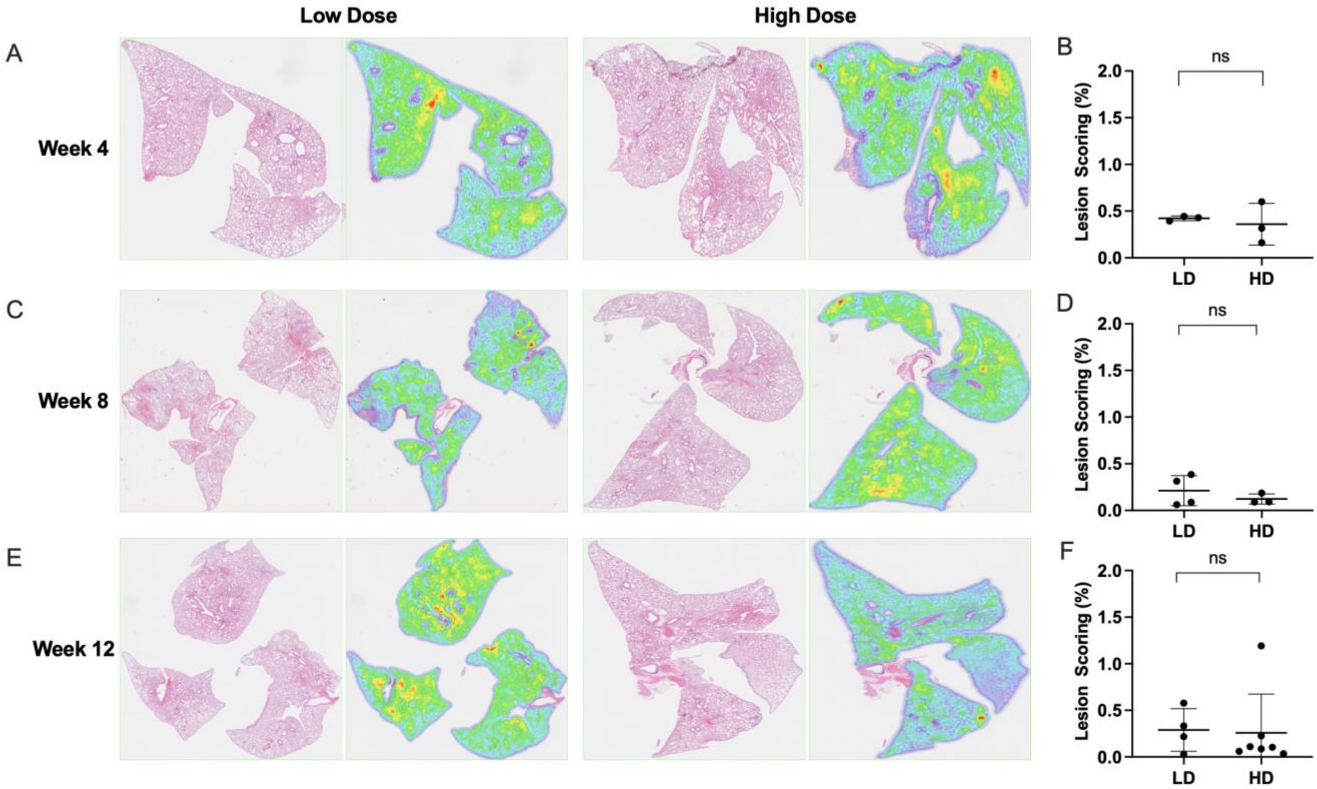

Figure S4: Histological lung changes in C3HeB/FeJ mice with MAC101 lung infection

Histological heat maps and lesion scoring of lungs at weeks 4 (A+B), weeks 8 (C+D) and weeks 12 (E+F) post-infection with low dose (LD) or high dose (HD) inoculum. Lesion scores were calculated as the proportion of infected area over the total lung area per animal (n=3-7 per group). Data displayed as mean + SD for each time point and analysed by unpaired test. Data representative of two studies with n=3-7 per time point. LD, low dose inoculum; HD, high dose inoculum; ns, not significant.
